# A Large Study About Reproductive Factors That Predict Hysterosalpingography-Identified Tubal Pathology: An Insight into the Necessity of Preconception Screening

**DOI:** 10.3390/jcm14010179

**Published:** 2024-12-31

**Authors:** Yurie Nako, Kuniaki Ota, Toshio Sujino, Junichiro Mitsui, Hisae Kamo, Shoko Katsumata, Yuko Takayanagi, Makiko Tajima, Tomonori Ishikawa, Akira Komiya, Kiyotaka Kawai

**Affiliations:** 1 Department of Reproductive Medicine, Kameda IVF Clinic Makuhari, Chiba 261-8501, Japan; 2 Department of Obstetrics and Gynecology, Kawasaki Medical School, Okayama 701-0192, Japan; 3 Fukushima Medical Center for Children and Women, Fukushima Medical University, Fukushima 960-1295, Japan; 4 Department of Comprehensive Reproductive Medicine, Graduate School of Medical and Dental Sciences, Institute of Science Tokyo, Tokyo 113-8519, Japan; 5 Department of Gynecology and Obstetrics, Sanmu Medical Center, Chiba 289-1326, Japan; 6 Perinatal and Maternal Medicine (Ibaraki), Graduate School of Medical and Dental Sciences, Institute of Science Tokyo, Tokyo 113-8519, Japan

**Keywords:** hysterosalpingography, infertility, tubal patency, positive CT-IgG, fibroids, endometrioma, painful defecation

## Abstract

**Background/Objectives**: Hysterosalpingography (HSG) is pivotal in delineating tubal pathology, but is associated with pain and exposure to ionizing radiation. This study investigated which reproductive factors predict HSG-identified tubal pathology. **Methods**: From May 2016 to August 2023, 3322 infertile females with HSG (mean age 33.9 ± 4.3 years) were assessed for fallopian tube status. **Results**: HSG indicated that 2764 had patent tubes while 558 (16.8%) had non-patent tubes. Unilateral and bilateral absence of free contrast spillage occurred in 377 (11.3%) and 181 (5.4%) cases, respectively. Non-spillage, denoted as non-patency, was seen in 148 (4.5%) and 153 (4.6%) right and left cases, respectively. Tubal occlusion was observed in 181 (5.4%) and 159 (5.4%) right and left cases, respectively. Hydrosalpinx was found in 37 (1.2%) right and 58 (1.7%) left cases. Multivariate logistic regression revealed CT-IgG positivity (odds ratio [OR]: 1.57), endometrioma (OR: 1.64), and fibroids (OR: 1.58) as independent factors for increased non-patency. CT-IgG positivity (OR: 1.92) and fibroids (OR: 1.88) were significant risk factors for occlusion. Painful defecation (OR: 2.79), CT-IgA positivity (OR: 2.09), CT-IgG positivity (OR: 2.07), and endometrioma (OR: 3.11) were significant risk factors for hydrosalpinx. **Conclusions**: In females with painful defecation, CT-IgG positivity, endometrioma, and fibroids, HSG may be used as a second-line investigation, with laparoscopy as the preferred assessment tool.

## 1. Introduction

Infertility, defined as the inability to conceive within 12 months of unprotected intercourse, affected an estimated 48.5 million couples globally in 2010 [1,2]. A fertility workup encompasses medical investigations to identify the various underlying causes of infertility in both males and females. While male-factor infertility is primarily a relative lack of functional sperm, female infertility may arise from ovulation disorders, diminished ovarian reserve, tubal factor infertility, or uterine factor infertility [3].

Tubal factor infertility is a major cause of female infertility, with a prevalence ranging between 11% and 30%. This condition commonly arises from infections (such as sexually transmitted infections like chlamydia and gonorrhea), prior pelvic surgery, peritonitis, or endometriosis [4,5,6,7,8]. Nevertheless, tubal integrity cannot be assumed by the lack of a history of pelvic inflammation or reproductive system diseases. For these reasons, the thorough evaluation of tubal integrity (including the visualization of obstructions or inefficient tubal function) has become a standard part of the basic infertility workup and represents a guide to the clinical management of infertile couples. Consequently, a tubal patency test has been routinely administered, since a part of the fertility workup is evaluating the risk of tubal pathology. Hysterosalpingography (HSG) using radiographs is less invasive than laparoscopy, and is often used for this purpose [3]. However, it is frequently described as painful, and involves exposure to ionizing radiation. Despite these drawbacks, HSG remains the first-choice test for ruling out tubal pathology in a fertility workup [9,10].

Conversely, the diagnostic accuracy of HSG, as determined by conventional meta-analysis, has shown a sensitivity of 65% and a specificity of 83% [11]. Consequently, the use of HSG as a first-line diagnostic tool has declined in recent years. Understanding the clinical predictors for an abnormal HSG could help decide who should undergo the test and who should not.

This study aimed to identify which patient characteristics and other infertility screening tests are most strongly associated with tubal pathology by performing a multivariable logistic regression analysis within a case-series study framework.

## 2. Materials and Methods

### 2.1. Patients

This cross-sectional study was designed in accordance with the Standards for Reporting of Diagnostic Accuracy Studies guidelines [12]. A total of 3322 reproductive-aged females with infertility who presented at the Kameda IVF clinic and underwent a tubal patency test as part of their evaluation of causal factors for female infertility between May 2016 and August 2023 were retrospectively analyzed. The study included both primary and secondary infertile females. The World Health Organization defines primary infertility as the inability to conceive after 1 year of sexual intercourse and secondary infertility as the inability to conceive following a previous pregnancy [13].

Initial medical records provided data on patient metrics, such as body mass index (BMI), and symptoms, such as genito–pelvic pain/penetration disorder, painful defecation, and dysmenorrhea. Diagnoses of benign tumors, such as fibroids, ovarian cysts, and endometriomas, were made using transvaginal ultrasound or magnetic resonance imaging. Fibroids were categorized according to the International Federation of Gynecology and Obstetrics classification system as submucosal, equivalent to types 0–2, and intramural fibroids, equivalent to type 3 [14]. Baseline serum levels of follicle-stimulating hormone (FSH), luteinizing hormone (LH), and estrogen on day 3 of the menstrual cycle, as well as serum progesterone during the luteal phase, serum anti-Müllerian hormone (AMH), serum thyroid-stimulating hormone (TSH), and serum free thyroxine (FT4), were analyzed as screening tests for infertility. Immunoglobulin (Ig)G and IgA antibodies to *Chlamydia trachomatis* (CT-IgG and CT-IgA) were also tested during the initial visit. Patients testing positive for these antibodies before HSG were treated orally with azithromycin at 1 mg daily. The exclusion criteria are patients who are clearly predicted to have tubal lesions and patients with cervical canal occlusion, although all patients undergo screening for infertility.

### 2.2. HCG Testing and Evaluation

HSG tests were performed within the first 10 days of the follicular phase, following the complete cessation of menstrual bleeding. A standard protocol was used, involving the infusion of a 5–10 cc water-based contrast medium into the uterine cavity and fallopian tubes using a balloon catheter (4.7 Fr 230 mm Trial EC-PRO Catheter; Kitazato Corp., Tokyo, Japan). Radiographs were taken to assess the uterine cavity and fallopian tube patency during the infusion of contrast medium. The process typically took approximately 5 min for image acquisition.

**Figure 1:**
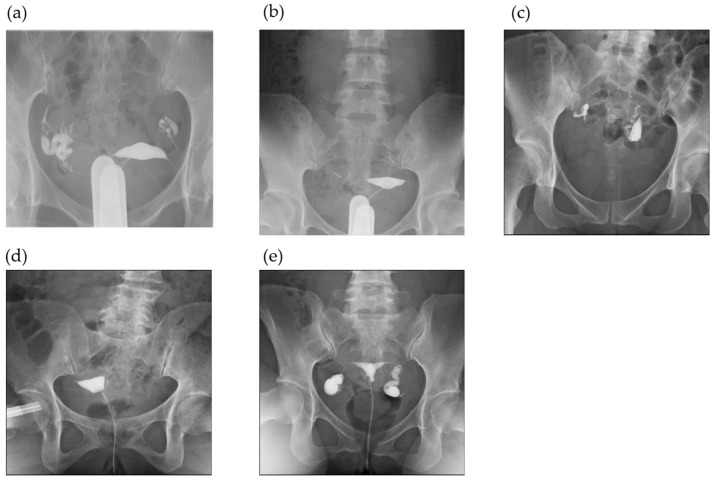
Diagnosis of fallopian tube patency, occlusion, and hydrosalpinx by hysterosalpingography. (**a**) Bilateral fallopian tube patency; (**b**) bilateral fallopian tube suboptimal patency; (**c**) the water-based medium remains; (**d**) bilateral tube occlusion; (**e**) bilateral tube hydrosalpinx.

Based on the images, tubal patency was categorized as follows: patency and non-patency, including suboptimal patency, occlusion, and hydrosalpinx. Patency was defined as visible contrast-medium spillage from the fimbria surrounding the ovary and collecting in the pelvis. Tubal patency was defined as the medium from the fimbria ending, described as fluid flow surrounding the ovary and its diffusion in the pelvis (Figure 1a). Tubal suboptimal patency involved delayed diffusion of the contrast medium in the pelvis or the contrast agent remaining (Figure 1b,c). Tubal occlusion was defined as the absence of any spillage (Figure 1c), and hydrosalpinx followed criteria involving partial tube filling at minimum or tube swelling without contrast appearing in the peritoneal cavity [15] (Figure 1d). Certified gynecologists performed all the procedures, and they determined the results of HSG.

### 2.3. Statistical Analyses

Statistical analyses were conducted using EZR (Saitama Medical Center, Jichi Medical University, Saitama, Japan), a graphical user interface for R 4.2(The R Foundation for Statistical Computing, Vienna, Austria) [16]. Variables were presented as mean ± standard deviation, and categorical variables were described as numbers and percentages. Logistic regression analyses were performed to evaluate HSG results without patency, employing forward stepwise methodology to identify independent predictive factors. Specifically, odds ratios (ORs) and 95% confidence intervals (CIs) were calculated in the multivariate analyses, controlling simultaneously for potential confounders, such as age, BMI, gravidity, and parity. Statistical significance was set at *p* < 0.05.

### 2.4. Ethical Approval

This study was approved by the Institutional Review Board of Kameda Medical Center and Kameda IVF Clinic, Makuhari (22-110). The study adhered to the ethical principles for medical research involving human subjects, as outlined in the Helsinki Declaration [17]. Informed consent was obtained in an opt-out manner on the website.

## 3. Results

### 3.1. Patient Characteristics

We evaluated 3322 infertile females who underwent HSG. Their main characteristics are detailed in Table 1. The mean age of the patients was 33.9 ± 4.3 years, with an age range of 23 to 47 years. The mean gravidity, parity, and number of miscarriages were 0.5 ± 0.8, 0.3 ± 0.5, and 0.2 ± 0.5, respectively. Serum levels of FSH, LH, and estrogen on day 3 of the menstrual cycle were 7.8 ± 3.5 IU/mL, 6.4 ± 2.8 IU/mL, and 38.3 ± 23.3 pg/mL, respectively. The mean BMI was 21.6 ± 3.5 kg/m^2^. Progesterone levels in the luteal phase and AMH were 16.7 ± 12.8 ng/mL and 4.1 ± 3.2 ng/mL, respectively. TSH and FT4 levels were 1.7 ± 2.2 μIU/mL and 1.3 ± 0.3 ng/dL, respectively.

Among the evaluated patients, 307/3322 (9.2%) were positive for CT-IgA, and 432/3322 (13.0%) were positive for CT-IgG. The prevalence rates for dysmenorrhea, genito–pelvic pain/penetration disorder, and painful defecation were 332/3322 (10.0%), 103/3322 (3.1%), and 1375/3322 (41.4%), respectively. Regarding imaging for infertility screening, the prevalence of submucosal fibroids, intramural fibroids, large fibroids (>4 cm), adenomyosis, and anomalies was 1.2% (41/3322), 3.8% (123/3322), 2.6% (87/3322), 2.8% (94/3322), and 3.2% (105/3322), respectively. Additionally, ovarian cysts and endometriomas were diagnosed in 1.8% (61/3322) and 5.1% (169/3322) of the cases.

### 3.2. HSG Findings

The tubal pathology of the 3322 infertile females is detailed in Table 1. In 2764 (83.2%) cases, HSG indicated tubal patency, while 558 (16.8%) were diagnosed with non-patency. Specifically, unilateral and bilateral absence of free contrast-material spillage was observed in 377 (11.3%) and 181 (5.4%) cases, respectively. Non-spillage of contrast material, indicating tubal suboptimal patency, was seen in 148 (4.5%) and 153 (4.6%) cases on the right and left sides, respectively. Tubal occlusion was observed in 181 (5.4%) and 159 (5.4%) cases on the right and left, respectively. Hydrosalpinx was found in 37 (1.2%) and 58 (1.7%) cases on the right and left sides, respectively.

### 3.3. Multivariate Analysis

A multivariate logistic regression analysis revealed that CT-IgG positivity (OR: 1.57, 95% CI: 1.17–2.11, *p* < 0.01), endometrioma (OR: 1.64, 95% CI: 1.12–2.41, *p* < 0.05), and fibroids (OR: 1.58, 95% CI: 1.09–2.29, *p* < 0.05) were independent factors for increased tubal non-patency (Table 2). This analysis also focused on factors affecting bilateral tubal non-patency, identifying CT-IgG positivity (OR: 1.82, 95% CI: 1.15–2.87, *p* < 0.05) and endometrioma (OR: 2.00, 95% CI: 1.13–3.56, *p* < 0.05) as independent contributors (Table 2).

Other significant risk factors identified through multivariate logistic regression were endometrioma for tubal suboptimal patency (OR: 2.15, 95% CI: 1.34–3.47, *p* < 0.01), and CT-IgG positivity (OR: 1.92, 95% CI: 1.33–2.78, *p* < 0.001) and fibroids (OR: 1.88, 95% CI: 1.21–2.94, *p* < 0.01) for tubal occlusion. Lastly, painful defecation (OR: 2.79, 95% CI: 1.06–7.32, *p* < 0.05), CT-IgA positivity (OR: 2.09, 95% CI: 1.04–4.13, *p* < 0.05), CT-IgG positivity (OR: 2.07, 95% CI: 1.09–3.99, *p* < 0.05), and endometrioma (OR: 3.11, 95% CI: 1.51–6.38, *p* < 0.01) were significant risk factors for tubal hydrosalpinx (Table 2).

## 4. Discussion

This study revealed that factors in infertile females influence the use of HSG as a screening test for infertility. Painful defecation, CT-IgG positivity, endometrioma, and fibroids were identified as predictive factors for tubal non-patency. HSG is an invasive procedure and is generally considered uncomfortable and painful, as 85% of females reported experiencing pain during the procedure, with half complaining of moderate to severe pain [18,19]. Therefore, our findings suggest that HSG should be recommended as a secondary screening test for infertility in cases of CT-IgG positivity, fibroids, endometrioma, and painful defecation, to minimize pain and exposure to ionizing radiation.

Once bilateral tubal patency is confirmed via HSG, it is reasonable to assume that patients have a low incidence of infertility due to tubal factors. This is because tubal disorders account for between 25% and 35% of all cases of infertility [20]. Therefore, the classification of the degree of tubal patency by HSG is of critical clinical value; it can be classified as tubal patency, non-patency, suboptimal patency, occlusion, and hydrosalpinx [21,22,23].

Ultrasound examination has limitations in differentiating the severity of hydrosalpinx, although applying a multidose contrast agent improves the diagnostic capability of HSG for hydrosalpinx [24,25]. Hydrosalpinx primarily results from chronic inflammatory stimulation and manifests as dilation or expansion of the fallopian tube during distal tubal obstruction. The main risk factors include pelvic inflammatory disease, endometriosis, appendicitis, and previous pelvic or abdominal surgery [26,27,28,29].

Notably, chlamydia infections can cause fallopian tube inflammation, leading to progressive pelvic floor adhesions, tubal non-patency, and hydrosalpinx [30,31,32]. The role of sexually transmitted infections in fertility impairment is well-documented; for instance, *C. trachomatis* is 3.4-fold more likely to be associated with tubal infertility [33,34]. This study further corroborated this association as an independent factor for tubal infertility, as revealed through multivariate logistic regression analysis.

**Figure 2:**
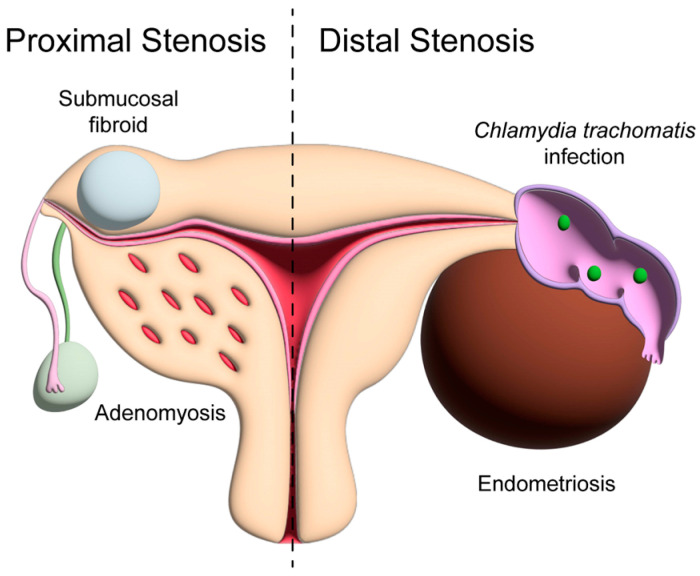
The causal factors of proximal- and distal-fallopian-tube obstruction, as elucidated in this study. Proximal-fallopian-tube obstruction is affected by the myometrial spasm via fibroids and adenomyosis (**left**), and distal fallopian- tube obstruction is affected by chlamydia infection and endometriosis (**right**).

Moreover, endometriosis, which occurs when ectopic endometrial tissue deposits outside of the uterus, particularly deep-infiltrating endometriosis and endometrioma, were found to be independent risk factors for tubal non-patency, including hydrosalpinx [35]. In our study, a multivariate logistic regression analysis revealed that CT-IgG positivity and endometrioma were independent risk factors for tubal non-patency, including tubal hydrosalpinx. At the initial screening for infertility, cases with a history of chlamydia infection and endometrioma were approximately two to three times more prone to tubal hydrosalpinx than those with tubal patency (Figure 2). Hence, such patients may be advised to undergo HSG as a secondary screening test for infertility.

Laparoscopic tubal plastic surgery is possible for tubal lesions caused by intra-abdominal adhesions in cases of a history of chlamydia. On the other hand, laparoscopic surgery can also be used to treat tubal disorders caused by endometriosis, but laparoscopic ovarian cystectomy for endometriomas may reduce ovarian reserve such as AMH [36,37]. Therefore, there is a problem of whether or not surgical intervention should be performed in cases of tubal non-patency. On the other hand, a method of preserving ovarian function by ethanol fixation has recently been reported [36], so such a strategy may be one option to prevent tubal non-patency from worsening.

Tubal evaluation includes HSG or saline-infused ultrasonography as noninvasive methods, and laparoscopy as an invasive method. The most visualized examination for tubal evaluation is laparoscopy [10,38], which allows direct visualization of tubal patency or pathology such as adhesions or phimosis using instilled dye. Some researchers have reported evaluating the accuracy of HSG compared with laparoscopy, with some showing discordance rates of up to 42–45% [39,40]. From a meta-analysis including 20 studies (mostly retrospective) and over 4000 patients with infertility, HSG had lower utility for ruling out tubal disease in the setting of tubal patency because of 65% sensitivity and 83% specificity for tubal patency [11]. The results of laparoscopy were more predictive of infertility than HSG in a prospective cohort study [10]. However, the American Society for Reproductive Medicine committee’s opinion states that evaluation should be “systematic, expeditious, and cost-effective, with initial emphasis on the least invasive methods for detecting the most common causes of infertility” [39]. Hence, laparoscopy, which requires surgical invasion, is avoided more than HSG. On the other hand, saline-infused ultrasonography as a non-invasive method allows for visualization of the contour of the uterine cavity and has the added benefit of evaluation for tubal patency. Sensitivity and specificity for saline-infused ultrasonography were 92% and 95%, respectively, and there were no significant differences between sensitivity or specificity in tubal assessment when directly comparing saline-infused ultrasonography and HSG [41]. However, the most significant factor favoring the selection of HSG over saline-infused ultrasonography for the initial evaluation of the female reproductive tract is its superiority in assessing tubal patency and pathology. Standard saline-infused ultrasonography can delineate whether at least one tube is open to allow the fluid to pass, but it cannot determine when both tubes are simultaneously open, as in HSG [42]. In addition, HSG also has an advantage over saline-infused ultrasonography, in that it can determine at which location within the tube an occlusion occurs. The architecture of the tube itself cannot be evaluated with saline-infused ultrasonography, and the tubes are not well delineated [43,44]. There is also a survey that shows that most physicians carry out HSG rather than saline-infused ultrasonography to evaluate tubal patency [45]. As a crucial point, a statement from the American Society for Reproductive Medicine Practice Committee in 2021 concluded that “HSG is the standard first-line test to evaluate tubal patency” [46].

Notably, our study demonstrated that CT-IgG positivity and fibroids were independent risk factors for tubal occlusion. When these findings were considered, the risk factor was approximately 2-fold higher for tubal occlusion compared to tubal patency. Proximal-tubal blockage, such as tubal occlusion, may be attributed to tubal spasm induced by elevated pressure from contrast injection [47]. A recent report described a uniquely identifiable spiral muscular ring contiguous with the adjacent myometrium surrounding the intramural part of the fallopian tube [48]. Therefore, spasms in the uterine myometrium, induced by disturbances such as fibroids and adenomyosis, may trigger spasms from the proximal tube to the distal tube, potentially leading to tubal occlusion (Figure 2).

Furthermore, we examined several uterine factors as potential risk factors for tubal occlusion. In our multivariate logistic regression analysis, uterine adenomyosis emerged as an independent factor for tubal occlusion (OR 2.09, 95% CI: 1.16–3.74, *p* < 0.05) (Supplementary Table S1). Given the spontaneous contractility of smooth myocytes in the myometrium, adenomyosis may lead to pelvic pain [49]. In cases of uterine adenomyosis, spasmodic contractions of the myometrium could induce tubal occlusion when a multidose contrast agent is injected into the uterine cavity.

Previous research has indicated that uterine fibroids may contribute to tubal infertility by locally compressing or occluding the tubal ostium, depending on the location of the fibroid [33]. Specifically, submucosal fibroids may directly obstruct the tubal ostium, leading to the diagnosis of tubal occlusion via HSG [50,51]. In our study, only submucosal fibroids were significant risk factors for tubal occlusion (OR 4.14, 95% CI: 1.93–8.86, *p* < 0.001), while intramural fibroids and fibroids >4 cm were not independently associated with this outcome (Supplementary Table S1). We suggest that patients undergo HSG reevaluation after resection of submucosal fibroids for a more accurate diagnosis of tubal patency, potentially mitigating the need for in vitro fertilization programs.

The key strengths of this study include the largest sample size, to the best of our knowledge, of patients using HSG as a screening test for infertility. Moreover, all procedures were conducted in a single center by experienced professionals, ensuring a real-world clinical setting. This consistency may minimize variables that might affect outcomes, such as differences in HSG technique and contrast volume (more contrast means more pain and less obstruction in HSG).

Nevertheless, this study had some limitations. First, we used a chlamydia antibody test to identify chlamydia infection. However, the presence of these antibodies does not specifically confirm chlamydia as the cause of the observed tubal pathology leading to infertility. Second, although a recent report indicated that the diagnostic values for assessing tubal patency via HSG were high, including high sensitivity, specificity, and negative predictive value [52], it is known that laparoscopy is the most available for diagnosing tubal pathology accurately [53,54]. However, only a limited number of cases were confirmed with laparoscopic procedures in our study. Finally, the single-center nature of the study could limit the generalizability of our findings. Therefore, future multi-center, prospective studies are warranted to confirm these findings.

## 5. Conclusions

HSG is a relatively inexpensive procedure, but it is an important diagnostic tool in the assessment of infertility because of the important role it plays in predicting the status of the fallopian tubes and the pelvis. On the other hand, HSG is painful and unavoidably leads to the application of ionizing radiation to the reproductive organs of young and potentially fertile females. In this study, we conducted an analysis to determine which infertile females should undergo HSG. Our findings suggest that HSG is advisable as a secondary screening test for infertility in females who exhibit symptoms such as painful defecation, who test positive for CT-IgG, or have conditions such as endometrioma and fibroids.

## Figures and Tables

**Table 1:** Study participant characteristics (*n* = 3322).

	Mean ± SD or Percentage
Age (y)	33.9 ± 4.3
Gravidity	0.5 ± 0.8
Parity	0.3 ± 0.5
Miscarriages	0.16 ± 0.5
BMI (kg/m^2^)	21.6 ± 3.5
Day 3 serum FSH (IU/mL)	7.8 ± 3.5 (Normal range: 3–10)
Day 3 serum LH (mIU/mL)	6.4 ± 2.8 (Normal range: 1.4–15)
Day 3 serum estrogen (pg/mL)	38.3 ± 23.3 (Normal range: 28.9–196.8)
Progesterone in the luteal phase (ng/mL)	16.7 ± 12.8 (Normal range: 5.0–30.8)
AMH (ng/mL)	4.1 ± 3.2
TSH (μIU/mL)	1.7 ± 2.2 (Normal range: 0.61–4.23)
FT4 (ng/dL)	1.3 ± 0.3 (Normal range: 0.75–1.45)
CT-IgA	307 (9.2%)
CT-IgG	432 (13.0%)
Complaint	
Genito–pelvic pain/penetration disorder	332 (10.0%)
Painful defecation	103 (3.1%)
Dysmenorrhea	1375 (41.4%)
Imaging with transvaginal ultrasound or MRI	
Uterine factor	
Fibroids	5.3% (177)
Submucosal fibroids	1.2% (41)
Intramural fibroids	3.8% (126)
Large fibroids (>4 cm)	2.6% (87)
Adenomyosis	2.8% (94)
Anomaly	3.2% (105)
Ovarian factor	
Cysts	1.8% (61)
Endometrioma	5.1% (169)
HSG	
Tubal patency	2764 (83.2%)
Tubal non-patency	558 (16.8%)
Unilateral	377 (11.3%)
Bilateral	181 (5.4%)
Right	
Suboptimal patency	148 (4.5%)
Occlusion	181 (5.4%)
Hydrosalpinx	37 (1.2)
Left	
Suboptimal patency	153 (4.6%)
Occlusion	159 (4.8%)
Hydrosalpinx	58 (1.7%)

BMI, body mass index; FSH, follicle stimulating hormone; LH, luteinizing hormone; AMH, anti-Müllerian hormone; TSH, serum thyroid-stimulating hormone; FT4, free thyroxine; CT-Ig, immunoglobulin antibodies to *Chlamydia trachomatis*; MRI, magnetic resonance imaging; HSG, hysterosalpingography.

**Table 2:** Univariate and multivariate logistic regression analysis for tubal pathology imaging with transvaginal ultrasound or MRI as an infertile screening (*n* = 3322).

	Crude OR	95% CI	*p*-Value	Adjusted OR ^a^	95% CI	*p*-Value
Tubal non-patency						
GPPPD	0.98	0.72–1.33	<0.001	0.88	0.62–1.25	NS
Painful defecation	1.36	0.84–2.20	<0.001	1.37	0.81–2.31	NS
Dysmenorrhea	1.06	0.89–1.28	<0.001	1.17	0.95–1.44	NS
Chlamydia IgA	1.69	1.28–2.24	<0.001	1.3	0.92–1.84	NS
Chlamydia IgG	1.75	1.37–2.22	<0.001	1.57	1.17–2.11	*p* < 0.01
Endometrioma	1.69	1.18–2.43	<0.001	1.64	1.12–2.41	*p* < 0.05
Fibroids	1.75	1.23–2.49	<0.001	1.58	1.09–2.29	*p* < 0.05
	data	data	data			
Tubal suboptimal patency						
GPPPD	0.95	0.62–1.48	NS	0.88	0.53–1.44	NS
Painful defecation	0.75	0.33–1.74	NS	0.71	0.29–1.70	NS
Dysmenorrhea	1.05	0.81–1.36	NS	1.11	0.83–1.49	NS
Chlamydia IgA	1.15	0.75–1.76	NS	1.29	0.78–2.16	NS
Chlamydia IgG	0.98	0.67–1.44	NS	0.92	0.58–1.46	NS
Endometrioma	2.14	1.36–3.37	<0.001	2.15	1.34–3.47	*p* < 0.01
Fibroids	1.33	0.79–2.23	NS	1.27	0.74–2.16	NS
Tubal occlusion						
GPPPD	0.91	0.60–1.37	NS	0.84	0.53–1.34	NS
Painful defecation	1.38	0.75–2.55	NS	1.58	0.79–3.11	NS
Dysmenorrhea	1.06	0.83–1.35	NS	1.23	0.93–1.62	NS
Chlamydia IgA	1.53	1.06–2.22	<0.05	1.04	0.66–1.63	NS
Chlamydia IgG	2.06	1.53–2.77	<0.001	1.92	1.33–2.78	*p* < 0.001
Endometrioma	0.86	0.48–1.53	NS	0.86	0.47–1.56	NS
Fibroids	2.24	1.48–3.38	<0.001	1.88	1.21–2.94	*p* < 0.01
Tubal hydrosalpinx						
GPPPD	0.87	0.40–1.91	NS	0.7	0.29–1.72	NS
Painful defecation	2.67	1.13–6.28	<0.05	2.79	1.06–7.32	*p* < 0.05
Dysmenorrhea	0.96	0.61–1.52	NS	0.85	0.50–1.43	NS
Chlamydia IgA	3.45	2.07–5.88	<0.001	2.09	1.04–4.13	*p* < 0.05
Chlamydia IgG	2.84	1.73–4.66	<0.001	2.07	1.09–3.99	*p* < 0.05
Endometrioma	3.16	1.64–6.09	<0.01	3.11	1.51–6.38	*p* < 0.01
Fibroids	0.7	0.22–2.23	NS	0.8	0.24–2.60	NS

GPPPD: genito–pelvic pain/penetration disorder, ^a^ adjusted for age, BMI, and the history of pregnancy and delivery.

## Data Availability

The datasets generated and/or analyzed during the current study are available from the corresponding author on reasonable request.

## Supplementary Materials

The following supporting information can be downloaded at: https://www.mdpi.com/article/10.3390/jcm14010179/s1, Table S1: Univariate and multivariate logistic regression analysis for tubal occlusion.
