# Supplementary material for: A Large Study About Reproductive Factors That Predict Hysterosalpingography-Identified Tubal Pathology: An Insight into the Necessity of Preconception Screening
Source: J Clin Med. 2024 Dec 31;14(1):179. doi: 10.3390/jcm14010179 (PMC11721101; doi:10.3390/jcm14010179)
Supplement: Supplementary file 1 [file jcm-14-00179-s001.zip › jcm-3366956-supplementary.pdf]

Supplemental Table S1. Univariate and multivariate logistic regression analysis for tubal occlusion.

	Crude OR	95% CI	P value	Adjusted OR*	95% CI	P value
Uterine factor						
Intramural fibroids	1.43	0.82-2.47	NS	1.04	0.56-1.94	NS
Submucosal fibroids	6.23	3.26-11.9	<0.0001	4.14	1.93-8.86	<0.0001
> 4 cm of Fibroid	1.81	12.5-2.60	<0.01	1.21	0.77-1.89	NS
Adenomyosis	2.19	1.26-3.79	<0.01	2.09	1.16-3.74	<0.05
Anomalies	1.48	0.82-2.68	NS	1.33	0.71-2.51	NS

*: adjusted for age, BMI, and the history of pregnancy and delivery
